# Preparing Medical Students for the Medical Interview

**Published:** 2017-02-15

**Authors:** Michael Kollhoff, C. Scott Owings, William Cathcart-Rake

**Affiliations:** University of Kansas School of Medicine-Salina, Salina, KS

**Keywords:** interview, medical history taking, behavior, communication, undergraduate medical education

## Introduction

The medical interview is possibly the most important encounter the physician has with a patient, frequently uncovering important clues as to what prompted the patient’s visit. To conduct an optimal medical interview, the physician must be aware of patient behavior patterns and be able to communicate effectively. In a seminal essay written in 1973, George Engel proclaimed, “The keystone around which medical care has evolved over the ages remains the interpersonal encounter between the patient and a physician.”^1^ He also claimed that the history obtained during the physician-patient encounter remains “the most sensitive and powerful instrument available to the physician.”^2^ Lipkin et al.^3^ argued that communication with patients is the core clinical skill for the practice of medicine. Given the importance of the physician-patient encounter and the medical interview, it is not surprising these topics are essential elements of the undergraduate medical education curriculum.

In 2005, the AAMC Task Force on the Clinical Skills Education of Medical Students published a monograph outlining recommendations for clinical skills curricula for undergraduate medical education.^4^ In 2008, the AAMC published a second monograph addressing the clinical skills curriculum and performance outcomes expected for pre-clerkship students.^5^ In both monographs, the task force members stressed the importance of medical students being able “to engage and communicate with a patient and to build a physician-patient relationship for the purposes of information gathering, guidance, education and support.”^4^, ^5^

Multiple authors have reported on the methods and successes of programs to enhance communication and medical interviewing skills.^6^–^11^ Although no one program seems to be superior to another, it was apparent that attention to development of these skills was important. The rural regional medical campus of University of Kansas School of Medicine-Salina (KUSM-S) designed a program entitled *Preparing Medical Students for the Medical Interview* that introduced the students to patterns of human behavior and effective communication techniques, information critical to starting the medical interview. The program introduced medical students to the basic interpersonal communication skills necessary to establish rapport with the patient during the initial moments of the medical interview, including a conversation template to follow for the first two minutes after entering the exam room. The students who completed this introductory program, delivered during the first two weeks of medical school, should be prepared to enter the exam room and create an environment that the patient immediately will perceive as safe enough to discuss their health issues.

The program was comprised of four learning activities: (1) Behavior Pattern Awareness (Social Styles), (2) Basic Listening Skills, (3) Recognizing Potential Interviewing Barriers, and (4) The First Two Minutes. This program helped the student navigate the new social encounters of the medical exam room and complemented the techniques of taking a history and performing a physical exam. The total time required to complete this program was approximately three hours, divided into three separate sessions. The first learning activity was covered in the first session. The second, third and fourth learning activities were covered in a second session. The elements of the fourth learning activity (The First Two Minutes) were practiced in a third session.

All social encounters between two individuals involve some degree of risk-taking. If one says “Hello” to a passerby, there is the risk that the person addressed will not respond in the manner expected by the initiator of the greeting. Similarly, when patients meet with a medical professional, especially during the first encounter, they must quickly decide if they can trust the care provider enough to take the risk of communicating their concerns. Patient-physician communication can be influenced by socioeconomic status, race, and gender.^12^–^15^ The intimate nature of the doctor-patient relationship, and the associated need for trust, requires that the patient be assured that the clinical environment is welcoming and safe. Meaningful dialogue, leading to an understanding of the patient’s health issues, making a diagnosis, and outlining a treatment program, start with a trusting relationship. The decision to trust is frequently made by the patient within seconds of a clinician entering the room.^16^

The KUSM-S program was developed to raise the students’ awareness of social styles and to introduce basic communication skills that foster empathy and trust. While some medical students may be cognizant of social styles and have developed effective communication skills prior to medical school matriculation, many of their peers were unaware of social styles and needed to hone their communication skills. This program allowed students to learn, or review, then practice effective communication skills, a set of skills that encompasses a variety of verbal and nonverbal techniques. The following four learning activities were designed to aid in acquisition and mastery of those skills.

## Learning Activity 1: Behavior Pattern Awareness

The initial step in preparing students for the medical interview was raising awareness of, or in some cases, introducing the student to the concept of Social Styles_®_ as outlined by the Tracom Group.^17^ Social Styles_®_ is a guide to discovery of predictable patterns of behavior, including how people interact with each other, the speed at which people do things, and other nonverbal types of body language. Using this model, behavior patterns are described as a combination of assertiveness (asking versus telling) and responsiveness (controlled feelings versus displaying feelings), resulting in four distinct social styles: (1) “driver”, more assertive (tells) and more controlled, (2) “analytical”, less assertive (asks) and more controlled, (3) “amiable”, less assertive (asks) and less controlled (emotive), and (4) “expressive”, more assertive (tells) and less controlled (emotive). Using a free online instrument, students completed a short survey of behaviors they see in themselves to identify their own social style.^18^ Students learned the nuances of their own behavior patterns and were challenged to become more cognizant of the fact that they are likely to be interviewing patients with behavior patterns different than their own. Students participated in a few simple exercises designed to raise awareness of the different behaviors inherent with each of the four Social Styles_®_, discussed the interpersonal issues that could arise as a result of miscommunication between individuals with different communication styles, and conceptualized ways to change one’s own communication style to be more receptive to others.

## Learning Activity 2: Basic Listening Skills

One of the attributes of physicians who receive high satisfaction ratings was being a good listener.^19^–^23^ It is essential that students learn to listen to their patients without interrupting them. In observational data from internal medicine and family medicine residents, Rhoades et al.^24^ found that resident physicians interrupted patients, on average, within 12 seconds after entering the room. Good listening does not imply that the clinician should ask the opening question, check a clock, wait until a certain amount of time has passed, then ask for clarification or introduce another question. However, there are a number of techniques that can be used to let the patient know that the doctor is interested and engaged in what is being said (i.e., actively listening). If practiced and done correctly, the patient should feel that the doctor is genuine, empathic, and shows them unconditional positive regard.

This learning activity introduced the student to several basic techniques that, when used effectively, can enhance the patient’s feeling of trust in the doctor. These listening skills were chosen for their value in helping the patient express the issues that brought them into the exam room that day, as well as helping the doctor listen to the things being said.^25^

1. **Attending** involves making eye contact, being a culturally comfortable distance from the person at or below their eye level, maintaining an open posture with nothing between the student and the patient, and leaning slightly toward them.
2. **Active Listening** encourages the student doctor to resist distractions and listen to the tone of the patient’s voice for cues to underlying feelings. The student is listening for basic themes that the patient is presenting while maintaining eye contact and correct posture.
3. **Encouraging** during the process of active listening lets the patient know on a more direct level that there is a connection by nodding when they finish a thought, or by giving small verbal encouragers like, “OK” or “tell me more.”
4. **Reflecting** through paraphrasing shows the patient that the student is listening actively in a slightly more aggressive manner. This is accomplished by repeating key phrases back to the patient, in their own words, for approval.
5. **Silence** is possibly the hardest skill of this group to master. Students, as well as patients, are often uncomfortable if something is not being verbalized continuously. However, if the student can remain attentive and quiet during patient silences, it gives the patient the message that what is being said is important and often will encourage them to carry their narrative to a higher level.

## Learning Activity 3: Recognizing Potential Interviewing Barriers

While the vast majority of patients seeking treatment are very willing to disclose their symptoms, there will be some patients that will be challenging to interview and call for more advanced interviewing techniques, as discussed in *Bates’ Guide to Physical Examination and History Taking*.^26^ Whether they are silent, angry, have behaviors that are offensive to the physician, or myriad other possibilities, it will be apparent soon after entering the exam room that the patient is not responding to the usual prompts. This is the student’s key to stop using the standard protocol and switch to more advanced techniques. Several commonly used counseling techniques were discussed with the students as possible approaches: acknowledging barriers immediately, responding to patient feelings as soon as they are noticed, giving affirmations, actively redirecting the patient if necessary, and the use of an objective, nonjudgmental voice during the interview. While in-depth study of these advanced techniques was outside the boundaries of the program, students discussed possible barriers to an optimal medical interview and some ways they might handle the barriers.

The individuals who present barriers or obstacles to a physician obtaining a history and physical exam often are called difficult or challenging patients. From a Social Styles_®_ perspective, they may be people who have behavior patterns different from those of the physician. Students were challenged to be aware of these differences and to consider ways they could modify their own behavior styles to communicate more effectively during challenging patient interviews. Adapting to the patient’s behavioral pattern may create the environment necessary for a patient to be heard and understood, leading to better treatment possibilities. Students also were encouraged to think about their personal philosophy of treatment as a guide in handling challenging situations.

## Learning Activity 4: The First Two Minutes

This learning activity was the culmination of the program and involved the preparation and actual mechanics of entering the exam room and concluded after listening to the patient’s concerns. While the medical interview will take longer than two minutes, the two minutes that occurred from the time the doctor entered the exam room to the conclusion of the patient’s initial description of reasons for the visit comprised the fourth learning activity: The First Two Minutes.

The activity consisted of six components, or steps, which when considered individually may not seem significant, but when considered as a whole helped the novice physician present himself or herself as a trusting and capable caregiver. Students were encouraged to commit these steps to memory, incorporating them into their ritual of conducting the medical interview.

1. **Read and Consider**. The student doctor reads the patient case file and spends a few moments considering information (e.g., reason for visit, age, gender, height, weight, and vital signs) that could be critical during the upcoming interview. This also aids in closure of a previous patient encounter and directs focus on the new one.
2. **Deep Breath and Smile.** This step encourages the clinician to take one or more deep cleansing breaths, which along with a sincere smile, provides stress reduction before every encounter. When coupled with positive thoughts, this creates an environment conducive to a helping relationship.^27^
3. **Knock and Enter.** The knock allows the patient time to prepare for someone to enter. It also may prevent a possible embarrassing situation for either physician or patient, if the physician was to enter unannounced. Additionally, if the doctor waits long enough to allow the patient to respond, it will provide some degree of empowerment to the patient. The door is opened and The First Two Minutes begins. Everything done up to this point is preparatory to entering the exam room, to make a good first impression, and to start building a trust relationship with the patient.
4. **Smile and Introduction.** Upon entering the room, the physician may have less than a second to no more than seven seconds to make a good impression.^16^, ^28^, ^29^ For this reason, wearing a comfortable or sincere smile is seen as critically important. The idea of a real smile can be juxtaposed with a fake smile often used by performers and sales personnel who know the importance of a smile but come off as insincere. Along with the smile, eye contact and a practiced introduction are essential. Montague et al.^30^ found that doctors who made eye contact with their patients and one or two social touches (e.g., handshake, hug, or pat on the back) were rated as more empathetic by their patients. Additionally, patients felt more connected to the doctor. The introduction may be the same for each individual patient. Something as simple as, “Hi, Mr. Jones, my name is Jim Smith. I am a student doctor at KU School of Medicine in Salina,” will suffice. Students are instructed to avoid asking the question, “How are you?” after the introduction. This question can lead to two unwanted results: the patient saying, “Fine,” when this is not the case or the patient immediately launching into a discussion of their chief complaint before the clinician is ready to listen.
5. **Wash and Weather.** Handwashing is one of the essential steps in preparing to conduct a physical exam but also can disconnect the patient from the doctor, even after the introduction. To ensure that the positive flow continues during the washing sequence, the clinician should garner some stock questions or stories to engage the patient while in transition. Asking about books or television shows the patient has read/watched recently or discussing the weather or sports are appropriate topics during this time. Bringing up controversial topics (e.g., politics, religion) is not a good idea, as it may lead to longer conversations than desired and can end up pitting the doctor’s opinions against the patient’s.
6. **Sit, Ask, and Listen.** During this step the doctor takes a position at or slightly below eye level of the patient and asks the opening question. This initial question should be rehearsed and can be the same for every patient encounter, something similar to, “How can I help you today?” is quite adequate. Sitting at, or a little below, the patient’s eye level transmits the message that the patient is in control.^31^ The student doctor continues by actively listening to the patient.

The First Two Minutes began with the opening of the exam room door and was not complete until the student had listened attentively to the patient for at least one minute. Within several days of introducing The First Two Minutes, KUSM-S students had the opportunity to practice these six steps on eight standardized patients. Students were instructed to listen to the patient’s response to the opening question using the listening skills from learning activity two. The goal during this phase was to allow the patient the time they need to describe, in their own words, their reason for coming in that day. Frequently, this important piece of patient empowerment is cut short by an overanxious clinician. Listening to the patient explain his or her symptoms is perhaps the most important part of the medical interview. The old adage that, if you listen to the patient they will tell you what is wrong, is sage advice. The medical history alone can lead to the final diagnosis in 76% of cases.^32^

## Discussion

Communication skills are a requisite part of being a physician. The physician who immediately can set his or her patient at ease and effectively engage the patient in a discussion should have a better chance of discovering what ails the patient and communicate a plan of action that the patient will accept. *Preparing Medical Students for the Medical Interview* was a program that the students at KUSM-S found valuable in preparing to interview their first patient, even if that patient was a role-playing standardized patient. Although we have not studied our results using stringent scientific principles, anecdotally, the students who have completed this brief program felt more comfortable and less nervous seeing their first patients than previous classes of students not exposed to the program.

## Conclusion

The importance of a positive physician-patient interaction in the initial stages of the medical interview cannot be overemphasized. KUSM-Salina’s *Preparing Medical Students for the Medical Interview* program was a relatively simple strategy to teach the beginning medical student how to approach the medical interview. The techniques helped medical students establish a trusting relationship with their patients and promoted effective communication, hopefully, resulting in improved patient care.
